# Double burden of malnutrition and obesity in children and adolescents from North Korean refugee families

**DOI:** 10.1371/journal.pone.0241963

**Published:** 2020-11-06

**Authors:** So-Young Kim, Seong-Woo Choi

**Affiliations:** Department of Preventive Medicine, Chosun University Medical School, Dong-gu, Gwangju, Republic of Korea; Yenepoya Medical College, Yenepoya University, INDIA

## Abstract

This study assessed the nutritional status of children and adolescents from North Korean refugee (NKR) families who have settled and are living in South Korea (SK). Among the 547 individuals who participated in the study, 526 were ultimately included after excluding 21 with missing height or weight data. Their nutritional status was estimated using the 2017 Korean National Growth Charts for children and adolescents. Stunting, underweight, wasting, and obesity were defined as a height-for-age z-score < −2.0, weight-for-age z-score < −2.0, weight-for-height z-score < −2.0, and body mass index z-score > 2.0, respectively. The overall prevalence of stunting, underweight, wasting, and obesity was 7.0%, 6.8%, 5.3%, and 9.1%, respectively. Meanwhile, the prevalence of stunting, underweight, wasting, and obesity was 5.4%, 7.0%, 7.6%, and 10.3% for individuals settled in SK for <5 years and 6.1%, 6.1%, 0.0%, and 13.3% for those living in SK for ≥5 years, respectively. Therefore, children and adolescents from NKR families experience the double burden of malnutrition and obesity.

## Introduction

Research on the health of migrants who have migrated from developing countries to developed countries is still ongoing [1,2]. However, the nutritional status and growth of children from migrant families is a poorly studied area. More than 30,000 North Korean refugees (NKRs) have entered South Korea (SK) since the 1990s owing to food shortage [3]. However, due to the special and complex reasons of NKRs, it is not known how many children of NKRs are currently growing up in SK.

Previous studies on NKRs have primarily focused on their settlement in SK [4,5], with relatively little research on their health and nutritional statuses. Furthermore, research on the health or nutritional statuses of NKRs has primarily focused on the period immediately after migration to SK [6–9]. To the best of our knowledge, only two studies have investigated the changes in the nutritional status of these individuals after settling in SK [10,11]. However, given that both studies included very few NK children and excluded those born in SK, only limited number of children and adolescents from NKR families have been evaluated for their nutritional status.

Therefore, the present study was conducted to evaluate the extent to which the nutritional status of children and adolescents from NKR families, including children born in SK, has improved.

## Materials and methods

### Subjects

This study included children and adolescents (aged <8 years) belonging to NKR families who have settled and are living in SK. The birth background of children and adolescents in NKR families can be divided into several categories [12], including those who were born in NK but have escaped NK and have entered SK; those who were born in a third country after one of the parents escaped from NK and subsequently entered SK; and those were born in SK after a parent settled in SK. Trained researchers explained the purpose of the study to the parents of these children and adolescents who were recruited through regional Hana Center, alternative schools, NGOs, etc. A survey was conducted from September 2017 to December 2019 using a structured questionnaire that included items on country of birth, duration of settlement, nationality of birth father and mother. Among the 547 children and adolescents who participated in this study, 21 were excluded due to missing height and weight data. Ultimately, 524 participants (275 males and 249 females) were included in this study. The institutional review board of Chosun University approved the study protocol (no. 2-1041055-AB-N-01-2017-0025), and written informed consent was obtained from participant’s aged more than 7 years and from the parents of those aged less than 7 years.

### Measures

Sex, age, country of birth, duration of settlement in SK, and nationality of the birth mother and father were determined. Height was measured using the mobile InKids height meter (InLab S50, InBody Co., Ltd., Seoul, Korea), which can measure heights of up to 200.0 cm at 0.1-cm intervals. Weight was measured using the CAS HE-58 scale (CAS, Gyeonggi-do, Korea), which provides measurements at 0.1-kg intervals. Body mass index (BMI) was calculated as the weight of the subject in kilograms divided by their height in meters squared.

### Nutritional status

The Korea Centre for Disease Control and Prevention and Korea Paediatrics Society developed the 2017 Korean National Growth Charts for children and adolescents [13] using the World Health Organization child growth standard [14] for children less than 3 years of age and data from the National Anthropometric Survey of 1997 (NAS1997) and 2005 (NAS2005) for those aged 3–18 years. The height-for-age (HAZ), weight-for-age (WAZ), weight-for-height (WHZ), and BMI-for-age (BMIZ) z-scores for each participant were calculated using the 2017 Korean National Growth Charts for children and adolescents.

### Statistical analysis

Statistical analysis was performed using the SPSS 23.0 software (IBM Co., Armonk, NY, USA). Categorical variables are presented as frequency and percentage and continuous variables as mean and standard deviation (SD). Stunting was defined as HAZ < −2 SD, underweight as WAZ < −2 SD, wasting as WHZ < −2 SD [15], and obesity as BMIZ > 2SD [16].

## Results

### Subject characteristics

The characteristics of the study subjects are summarized in Table 1. Among the participants, 52.5% were male and 47.5% were female, with a mean age of 132.9 ± 65.2 months. The mean HAZ, WAZ, WHZ, and BMIZ were −0.20 ± 1.32, −0.03 ± 1.34, 0.29 ± 1.51, and 0.08 ± 1.46, respectively. Among the participants, 45.6%, 37.5%, and 16.7% were born in China, SK, and NK, respectively. With regard to the duration of settlement in SK, 65.4% were settled in SK for <5 years, whereas 34.6% were settled in SK for ≥5 years.

**Table 1:** Characteristics of the study subjects.

Parameter	Total	Male	Female	P-value
Number	526 (100.0)	276 (52.5)	250 (47.5)	
Age (years)	11.1 ± 5.4	10.9 ± 5.4	11.2 ± 5.4	0.547
Height (cm)	138.3 ± 29.6	140.3 ± 31.4	136.2 ± 27.3	0.108
Weight (kg)	40.3 ± 20.3	41.9 ± 21.8	38.6 ± 18.3	0.062
BMI (kg/m^2^)	19.4 ± 4.1	19.5 ± 4.1	19.2 ± 4.0	0.417
HAZ	−0.20 ± 1.32	−0.16 ± 1.21	−0.24 ± 1.42	0.516
WAZ	−0.03 ± 1.34	−0.05 ± 1.41	−0.01 ± 1.26	0.749
WHZ	0.29 ± 1.51	0.21 ± 1.52	0.38 ± 1.50	0.217
BMIZ	0.08 ± 1.46	0.06 ± 1.54	0.10 ± 1.36	0.796
Country of birth				0.005
South Korea	193 (37.5)	100 (36.6)	93 (38.4)	
North Korea	86 (16.7)	33 (12.1)	53 (21.9)	
China	235 (45.6)	140 (51.3)	95 (39.3)	
Others	6 (1.2)	3 (1.1)	3 (1.2)	
Duration of settlement	4.2 ± 3.4	4.2 ± 3.3	4.1 ± 3.6	0.981
<5 years	185 (65.4)	96 (65.3)	89 (65.4)	
≥5 years	98 (34.6)	51 (34.7)	47 (34.6)	
Nationality of birth mother				0.194
South Korea	12 (2.3)	7 (2.6)	5 (2.0)	
North Korea	503 (96.5)	266 (97.1)	237 (96.0)	
China	6 (1.2)	1 (0.4)	5 (2.0)	
Nationality of birth father				0.003
South Korea	112 (21.6)	57 (21.0)	55 (21.0)	
North Korea	137 (26.4)	55 (20.2)	82 (33.3)	
China	265 (51.2)	158 (58.1)	107 (43.5)	
Others	4 (0.8)	2 (0.7)	2 (0.8)	

Values are presented as number (%) or mean ± standard deviation.

HAZ; Height-for-age z-score, WAZ; Weight-for-age z-score, WHZ; Weight-for-height z-score, BMIZ; BMI-for-age z-score.

### Prevalence of stunting, underweight, wasting, and obesity

As shown in Table 2, the prevalence of stunting, underweight, wasting, and obesity was 7.0%, 6.8%, 5.3%, and 9.1% respectively. On the other hand, the prevalence of stunting, underweight, wasting, and obesity was 6.9%, 8.3%, 6.2%, and 11.2% in males and 7.2%, 5.2%, 4.4%, and 6.8% in females, respectively.

**Table 2:** Prevalence of stunting, underweight, and wasting.

	Total	Male	Female	P-value
Stunting	37 (7.0)	19 (6.9)	18 (7.2)	0.887
Underweight	36 (6.8)	23 (8.3)	13 (5.2)	0.155
Wasting	28 (5.3)	17 (6.2)	11 (4.4)	0.359
Obesity	48 (9.1)	31 (11.2)	17 (6.8)	0.078

Values are presented as number (%).

Stunting is defined as a height-for-age z-score of <−2.0.

Underweight is defined as a weight-for-age z-score of <−2.0.

Wasting is defined as a weight-for-height z-score of <−2.0.

Obesity is defined as a BMI-for-age z-score of >2.0.

### Prevalence of stunting, underweight, wasting, and obesity according to the duration of settlement in SK

The prevalence of stunting, underweight, wasting, and obesity according to the duration of settlement is summarized in Table 3. Accordingly, the prevalence of stunting, underweight, wasting, and obesity was 5.4%, 7.0%, 7.6%, and 10.3% among individuals who had settled for <5 years and 6.1%, 6.1%, 0.0%, and 13.3% among those who had settled for >5 years, respectively. Furthermore, among those born in SK, the prevalence of stunting, underweight, and wasting was 8.3%, 6.7%, and 7.8%, respectively.

**Table 3:** Prevalence of stunting, underweight, and wasting according to the duration of settlement in SK.

	Country of birth			P-value
	North Korea or others		South Korea	
	Duration of settlements			
	<5 years	≥5 years		
Stunting	10 (5.4)	6 (6.1)	16(8.3)	0.516
Underweight	13 (7.0)	6 (6.1)	13(6.7)	0.959
Wasting	14 (7.6)	0 (0.0)	13(6.8)	0.024
Obesity	19 (10.3)	13 (13.3)	15(7.8)	0.323

Values are presented as number (%).

Stunting is defined as a height-for-age z-score of <−2.0.

Underweight is defined as a weight-for-age z-score of <−2.0.

Wasting is defined as a weight-for-height z-score of <−2.0.

Obesity is defined as a BMI-for-age z-score of >2.0.

### Prevalence of stunting, underweight, and wasting among subjects born in NK before and after 2000

**Fig 1:**
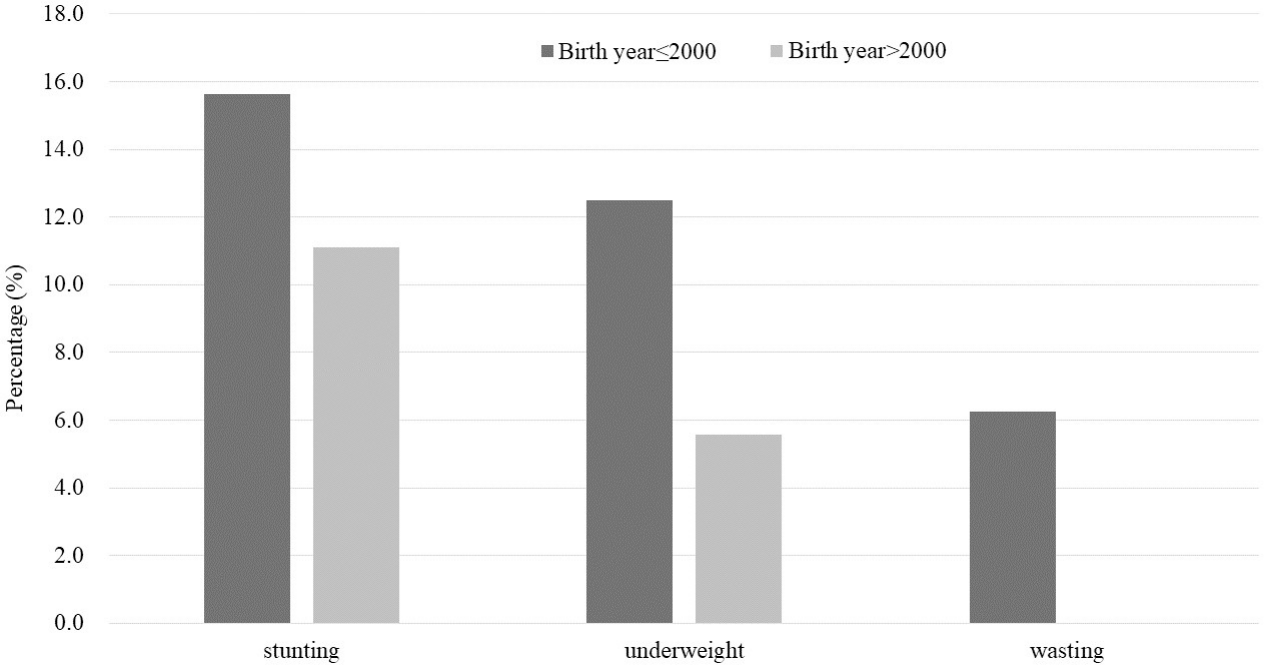
Prevalence of stunting, underweight, and wasting among individuals born in North Korea before and after 2000.

The prevalence of stunting, underweight, and wasting among individuals born in NK before and after 2000 is presented in Fig 1. Accordingly, the prevalence of stunting, underweight, and wasting was 15.6%, 12.5%, and 6.3% among those born in NK before 2000 and 11.1%, 5.6%, and 0.0% among those born in NK after 2000, respectively.

## Discussion

The present study evaluated the nutritional status of 526 children and adolescents in NKR families who had settled and are living in SK. Our results showed that the prevalence of stunting, underweight, and wasting among our participants was 7.0%, 6.8%, and 5.3%, respectively, with obesity also having been observed at a prevalence of 9.1%.

Most previous studies have evaluated the nutritional status of NKRs using health data immediately after entering SK, such as Hanawon health data [6–9]. As a result, severe growth retardation and the malnutrition status of NKRs were reported. Although many nutritional evaluations have been conducted in individuals immediately after they have entered SK, few studies have evaluated how their nutritional statuses have changed since they settled in SK. To the best of our knowledge, only two studies have assessed the changes in their nutritional status after settling in SK. In one study with 103 NKRs aged 12–24 years, the researchers compared them with sex- and age-matched 309 SK individuals [10]. They reported that NKRs had settled in SK for approximately 2 years, but they still were significantly shorter and leaner than SK subjects. Another study [11] investigated 70 NKRs aged 6–15 years who had settled in SK for approximately 2 years. Compared with Hanawon health data immediately after entering SK, their nutritional status showed improvement from 11.4% to 5.7% for stunting and 14.3% to 1.4% for underweight after settling in SK for approximately 2 years. Similarly, the present study found an improvement in nutritional status as the duration of settlement in SK increased, with the prevalence of underweight and wasting being 7.0% and 7.6% among individuals who had settled for less than 5 years and 6.1% and 0.0% among those who had settled for more than 5 years, respectively. Despite this improvement, their nutritional status is still considered serious. Even among children and adolescents in NKR families who were themselves born in SK, 8.3% exhibited stunting, 6.7% were underweight, and 6.8% had wasting. In such cases, malnutrition could not simply be attributed to the duration of settlement in SK but rather to the low socio-economic position of NKR families [17]. A previous study demonstrated that 51.4% and 41.4% of NKR reported being food insecure and severely food insecure, respectively [11]. In addition, we compared the nutritional status of children born before and after 2000, when the food shortage in North Korea was the worst. Our data showed that the prevalence of stunting, underweight, and wasting was 15.6%, 12.5%, and 6.3% among individuals born in NK before 2000 and 11.1%, 5.6%, and 0.0% among those born in NK after 2000, respectively (Fig 1).

One previous study using Hanawon data for NKRs who entered SK between 2009 and 2010 revealed that none of the children had a weight higher than the 97^th^ percentile [9]. However, the present study showed that the prevalence of obesity in NKR families was 9.1%. Moreover, another study reported that the prevalence of obesity was 1.4% using Hanawon data, which subsequently increased to 5.7% after settling in SK for approximately 2 years [11]. Shortly after NKRs entered SK, only malnutrition has remained a major health problem [9]. However, the prevalence of obesity has been increasing as the settlement period increases. Children and adolescents in NKR families who have settled and are living in SK now face the “double burden” of both under- and overnutrition [18]. Researchers have compared the nutritional intake status of NKR children aged 6–18 years with data for SK children from the 2009–2010 Korean National Health and Nutrition Examination Survey, randomly matched for age and gender. Their results showed that NKR children had higher rates of fat intake than SK children [19]. Insufficient food intake in early life may alter metabolic processes, possibly decreasing the use of fat as an energy source, thereby resulting in its accumulation in the body [20] and the increased risk of obesity [21,22]. Therefore, it is important to continuously assess the status of overweight and obesity as well as that of inadequate nutrition among children in NKR families.

One limitation of the present study was that the participants had not been randomized to children and adolescents in NKR families. As a result, the results cannot be generalized to all children and adolescents in NKR families. Moreover, no detailed information regarding household income and parents’ education levels was available, which could affect the nutritional statuses of children and adolescents. Nevertheless, this study remains significant considering that it provides data regarding the nutritional statuses of children and adolescents in NKR families who have entered and settled in SK.

## Conclusion

The present results demonstrate that children and adolescents in NKR families experience the double burden of malnutrition and obesity.

## Supporting information

S1 File (SAV)

## References

[1] Gadd M, Sundquist J, Johansson SE, Wändell P. Do immigrants have an increased prevalence of unhealthy behaviours and risk factors for coronary heart disease? European Journal of Cardiovascular Prevention & Rehabilitation 2005;12(6):535–541. 10.1097/01.hjr.0000174829.25388.ed 16319542

[2] McDonald JT, Kennedy S. Is migration to Canada associated with unhealthy weight gain? Overweight and obesity among Canada’s immigrants. Social Science & Medicine 2005;61(12):2469–2481. 10.1016/j.socscimed.2005.05.004 15972242

[3] Ministry of Unification. Current status of North Korean refugee entry. Sejong: Ministry of Unification; 2020 [cited 2020 26th, July]. https://www.unikorea.go.kr/unikorea/business/statistics.

[4] Baek HJ, Kil EB, Yoon IJ, Lee YR. A study on psychological adaptation of North Korean adolescent refugees in South Korea. Studies on Korean Youth 2007;18(2):183–211.

[5] Yoo GH, Bang ER, Han EJ. A case study on school achievement and social adaptation of North Korean refugee children & adolescents. Journal of Korean Home Management Association 2004;22(5):185–196.

[6] Pak S. The biological standard of living in the two Koreas. Economics & Human Biology 2004;2(3):511–521. 10.1016/j.ehb.2004.09.001 15576250

[7] Pak S. The growth status of North Korean refugee children and adolescents from 6 to 19 years of age. Economics & Human Biology 2010;8(3):385–395. 10.1016/j.ehb.2010.05.006 20646970

[8] Kim YY. An evaluation of the health status of children from North Korea. Health & Nursing 2005;17(2):55–63.

[9] Lee IS, Park HR, Kim YS, Park HJ. Physical and psychological health status of North Korean defector children. Journal of Korean Academy of Child Health Nursing 2011;17(4):256–263. 2011.17.4.256.

[10] Choi SK, Park SM, Joung H. Still life with less: North Korean young adult defectors in South Korea show continued poor nutrition and physique. Nutrition Research and Practice 2010;4(2):136–141. 10.4162/nrp.2010.4.2.136 20461202 PMC2867224

[11] Lee SK, Nam SY, Hoffman D. Changes in nutritional status among displaced North Korean children living in South Korea. Annals of Human Biology 2015;42(6):581–584. 10.3109/03014460.2014.993704 25977217

[12] Lee KY, Kim MK. Diversity of youths with a background of defection from North Korea: assistance policy analysis and implications. The Journal of Northeast Asia Research 2015;30(2):93–129.

[13] Kim JH, Yun S, Hwang SS, Shim JO, Chae HW, Lee YJ, et al The 2017 Korean National Growth Charts for children and adolescents: development, improvement, and prospects. Korean Journal of Pediatrics 2018;61(5):135–149. 10.3345/kjp.2018.61.5.135 29853938 PMC5976563

[14] World Health Organization. WHO child growth standards: length/height-for-age, weight-for-age, weight-for-length, weight-for-height and body mass index-for-age: methods and development. Geneva: World Health Organization; 2006.

[15] de Onis M, Blössner M. The World Health Organization global database on child growth and malnutrition: methodology and applications. International Jouranl of Epidemiology 2003;32(4):518–526. 10.1093/ije/dyg099 12913022

[16] de Onis M, Onyango AW, Borghi E, Siyam A, Nishida C, Siekmann J. Development of a WHO growth reference for school-aged children and adolescents. Bulletin of the World Health Organization 2007;85:660–667. 10.2471/blt.07.043497 18026621 PMC2636412

[17] Korea Hana Foundation. Settlement Survey of North Korean refugees in South Korea. Seoul: Korea Hana Foundation; 2019.

[18] Wells JC, Sawaya AL, Wibaek R, Mwangome M, Poullas MS, Yajnik CS, et al The double burden of malnutrition: aetiological pathways and consequences for health. The Lancet 2020;395(10217):75–88. 10.1016/S0140-6736(19)32472-9 31852605 PMC7613491

[19] Lee SK, Nam SY. Comparison of food and nutrient consumption status between displaced North Korean children in South Korea and South Korean children. Korean Journal of Community Nutrition 2012;17(4):407–418. 10.5720/kjcn.2012.17.4.407

[20] Hoffman DJ, Sawaya AL, Verreschi I, Tucker KL, Roberts SB. Why are nutritionally stunted children at increased risk of obesity? Studies of metabolic rate and fat oxidation in shantytown children from São Paulo, Brazil. The American Journal of Clinical Nutrition 2000;72(3):702–707. 10.1093/ajcn/72.3.702 10966887

[21] Sawaya AL, Martins P, Hoffman D, Roberts SB. The link between childhood undernutrition and risk of chronic diseases in adulthood: a case study of Brazil. Nutrition Reviews 2003;61(5 Pt 1):168–175. 10.1301/nr.2003.may.168-175 12822705

[22] Popkin BM, Richards MK, Montiero CA. Stunting is associated with overweight in children of four nations that are undergoing the nutrition transition. The Journal of Nutrition 1996;126(12):3009–3016. 10.1093/jn/126.12.3009 9001368

